# Molecular and Metabolic Reprogramming: Pulling the Strings Toward Tumor Metastasis

**DOI:** 10.3389/fonc.2021.656851

**Published:** 2021-06-03

**Authors:** Ana Hipólito, Filipa Martins, Cindy Mendes, Filipa Lopes-Coelho, Jacinta Serpa

**Affiliations:** ^1^ CEDOC, Chronic Diseases Research Centre, NOVA Medical School|Faculdade de Ciências Médicas, Universidade NOVA de Lisboa, Lisboa, Portugal; ^2^ Unidade de Investigação em Patobiologia Molecular (UIPM), Instituto Português de Oncologia de Lisboa Francisco Gentil (IPOLFG), Lisboa, Portugal

**Keywords:** metabolic reprogramming, metastasis, metastatic cascade, tumor microenvironment, new therapies

## Abstract

Metastasis is a major hurdle to the efficient treatment of cancer, accounting for the great majority of cancer-related deaths. Although several studies have disclosed the detailed mechanisms underlying primary tumor formation, the emergence of metastatic disease remains poorly understood. This multistep process encompasses the dissemination of cancer cells to distant organs, followed by their adaptation to foreign microenvironments and establishment in secondary tumors. During the last decades, it was discovered that these events may be favored by particular metabolic patterns, which are dependent on reprogrammed signaling pathways in cancer cells while they acquire metastatic traits. In this review, we present current knowledge of molecular mechanisms that coordinate the crosstalk between metastatic signaling and cellular metabolism. The recent findings involving the contribution of crucial metabolic pathways involved in the bioenergetics and biosynthesis control in metastatic cells are summarized. Finally, we highlight new promising metabolism-based therapeutic strategies as a putative way of impairing metastasis.

## Introduction

**Figure 1:**
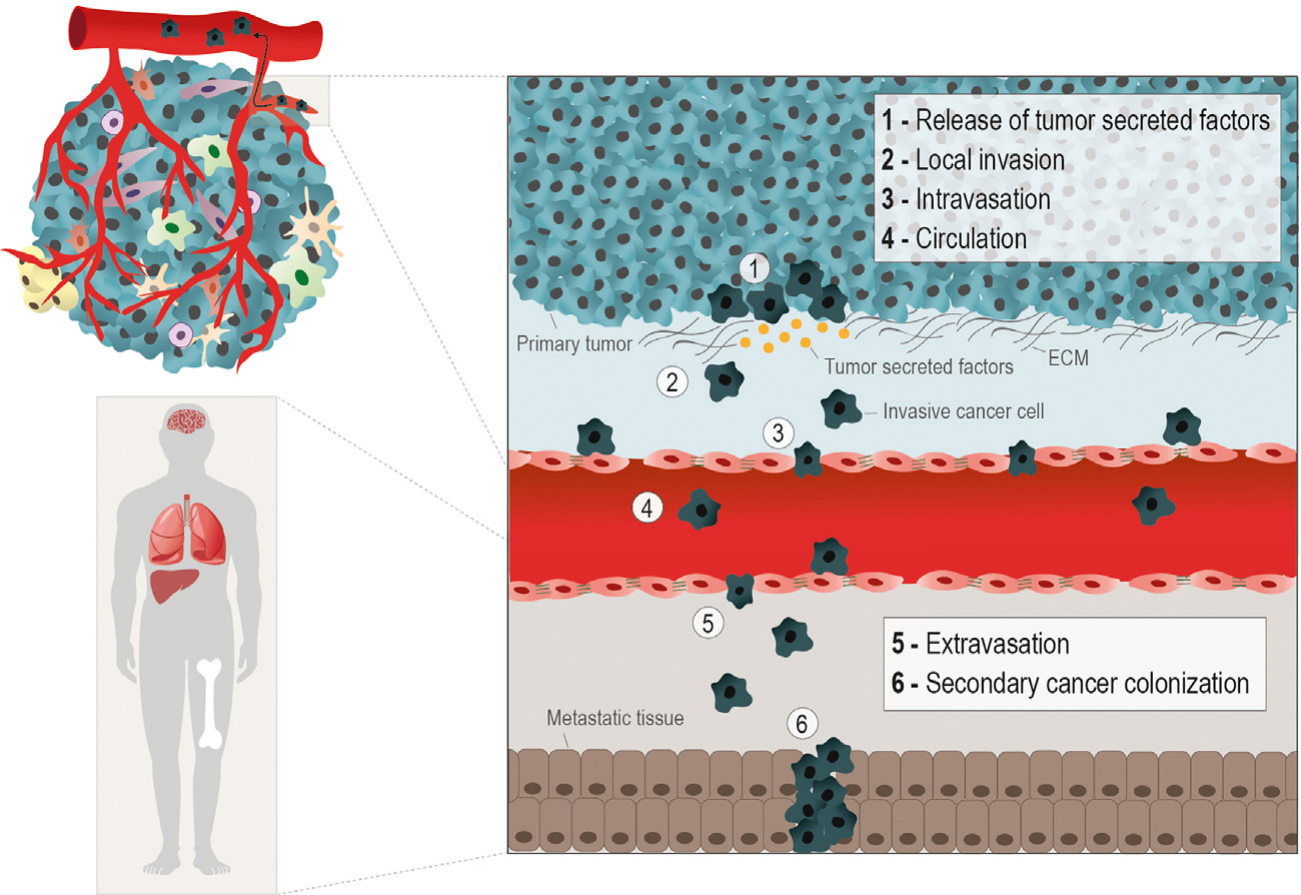
Hematogenous metastatic route. The metastatic cascade starts with molecular and morphological changes in cancer cells, enabling their release from the primary tumor. The production of tumor secreted factors (*e.g.* MMPs) by cancer cells to degrade the extracellular matrix (ECM) (1), helps the gain of migratory and invasive abilities (2). The metastasizing cancer cells, depending on the cancer type, rely on epithelial to mesenchymal transition (EMT), in which cancer cells acquires a mesenchymal phenotype through the loss epithelial cell-cell contacts, as E-Cadherin, to facilitate the penetration into the basal membrane. However, in other scenarios collective or cluster‐based migration and invasion can contribute to cancer cell intravasation independent of EMT. The different phases of the hematogenous metastatic cascade are: local invasion (1 and 2); intravasation into vessels (3); circulation into the bloodstream (4); extravasion into a distant organ or tissue (5), and the formation of a secondary tumor in the metastatic niche (6). The metabolic remodeling during the metastatic processes is crucial for the efficient colonization of cancer cells in distant sites, typically nutrient and oxygen-rich areas, as lungs, liver, bones and brain.

Metastasis is the main critical issue in cancer progression, being responsible for 90% of cancer-related deaths (1). The way cancer cells manage to detach from the primary tumor, migrate, invade, and follow the metastatic routes (*e.g.* hematogenous, lymphatic, serous, direct and nervous) depends on several forces and stresses. The transformation a cancer cell undergoes to get free from the tumor and gain migratory and invasive capacity, requires a multitude of orchestrated molecular changes (2). The most well-known metastatic route is the hematogenous and carcinomas, which account for more than 80% of all the malignant neoplasias, have been the most studied in the metastatic context (3). This metastatic cascade is composed by the following steps: cancer cells release from the primary tumor, local invasion, vessels intravasation, trip on the blood circulation, vessels extravasation, and secondary organs colonization (4) (**Figure 1**).

The epithelial-to-mesenchymal transition (EMT) in cancer cells is an important event to allow these cells to invade and intravasate the vasculature. The morphological alterations necessary for a static carcinoma cell to become migratory involve the replacement of molecules characteristic of epithelial architecture by mesenchymal molecules. The best known EMT markers are the loss of E-cadherin expression, an epithelial adhesion molecule (adherens junction), and the gain of vimentin expression, a protein from the intermediate filaments of the cytoskeleton (5, 6). The metastatic capacity of cancer cells must be addressed considering the molecular changes exhibited by the cancer cell, and the composition and physical properties of the substrate (matrix) to be invaded. The local invasion capacity is crucial in the metastatic cascade since it allows detached cancer cells to infiltrate the stroma and enter the bloodstream (7). Invadopodia are subcellular F-actin-rich structures present in invasive cancer cells, composed of several signaling, cytoskeletal, adhesion and matrix degradation proteins, that are mainly responsible for offering traction for these cells and degrading the ECM (8, 9) (**Figure 1**). The ECM proteins play an important role as a network for sharing signaling molecules and (in)organic compounds and as a supportive scaffold for the organization of cells within tissues and organs (10, 11). However, ECM proteins are themselves an important complex of insoluble ligands involved in signaling activation, mainly through the interaction with integrins (12). The stiffness and resistance of stromal ECM are dependent on the relative composition of ECM protein types (3, 13, 14). Our team and others have published that certain ECM proteins, such as fibronectin and different types of collagen and laminin, can favor the cancer metastatic process (15–18).

Intravasation is defined as the ability of cancer cells to enter the vessels and follow the bloodstream (4, 19, 20). Once in the bloodstream, cancer cells face a hostile environment and are exposed to mechanical stress, shear forces, and to the presence of immune cells and oxidative stress, which may compromise their successful arrival at the distant metastasis site (21, 22). As an escape to those adversities, cancer cells establish a vital partnership with platelets during this transition. Cancer cells release soluble factors and activate platelets, which sheath them in a clot, impeding cytolysis and assuring their protection against adversities (23). Platelets by themselves have pro-metastatic effects by inducing EMT-like status and increase cancer cell invasive ability and extravasation (22, 24, 25). Therefore, the next tricky step of the metastatic cascade is the extravasation of these cells from the vessels into the nearby tissue (21). This process depends on the adhesion of these cells to endothelium and modulation of the endothelial barrier, promoting their extravasation across the vessels’ wall. Usually, tumor cells get physically arrested in small capillaries, where they migrate across the endothelial barrier. This occurs by transendothelial migration, either by paracellular migration, in which tumor cells cross between adjacent endothelial cells, leading to cellular rearrangements; or by transcellular transendothelial migration, in which tumor cells migrate directly across the endothelial cell body (21, 26). During this process, tumor-specific proteins, including cadherins, integrins and selectins, amongst other molecules responsible for the adhesion to endothelium have a central role (27, 28). Tumor cells that resisted all previous steps and managed to survive and colonize a secondary organ, eventually form distant metastases. Successful colonization depends on the adaptation and positive interaction of the tumor cell and the organ’s microenvironment (29). Tumor cells must rely on a permissive microenvironment to colonize and, thus, the specific cellular traits may influence the adaptation of tumor cells to this microenvironment and metabolic remodeling certainly plays a role in this process.

The metabolic adaptation to the microenvironment is crucial for a primary tumor to grow locally, invade and metastasize. The metastatic cancer cells are especially more plastic than non-metastatic cancer cells, since the latter ones must survive in the microenvironment where the primary tumor develops and in the microenvironment of the secondary organ to which they metastasize.

## Rewired Metabolic Traits in Metastasis, a Way of Surviving Outside of the Primary Tumor

**Figure 2:**
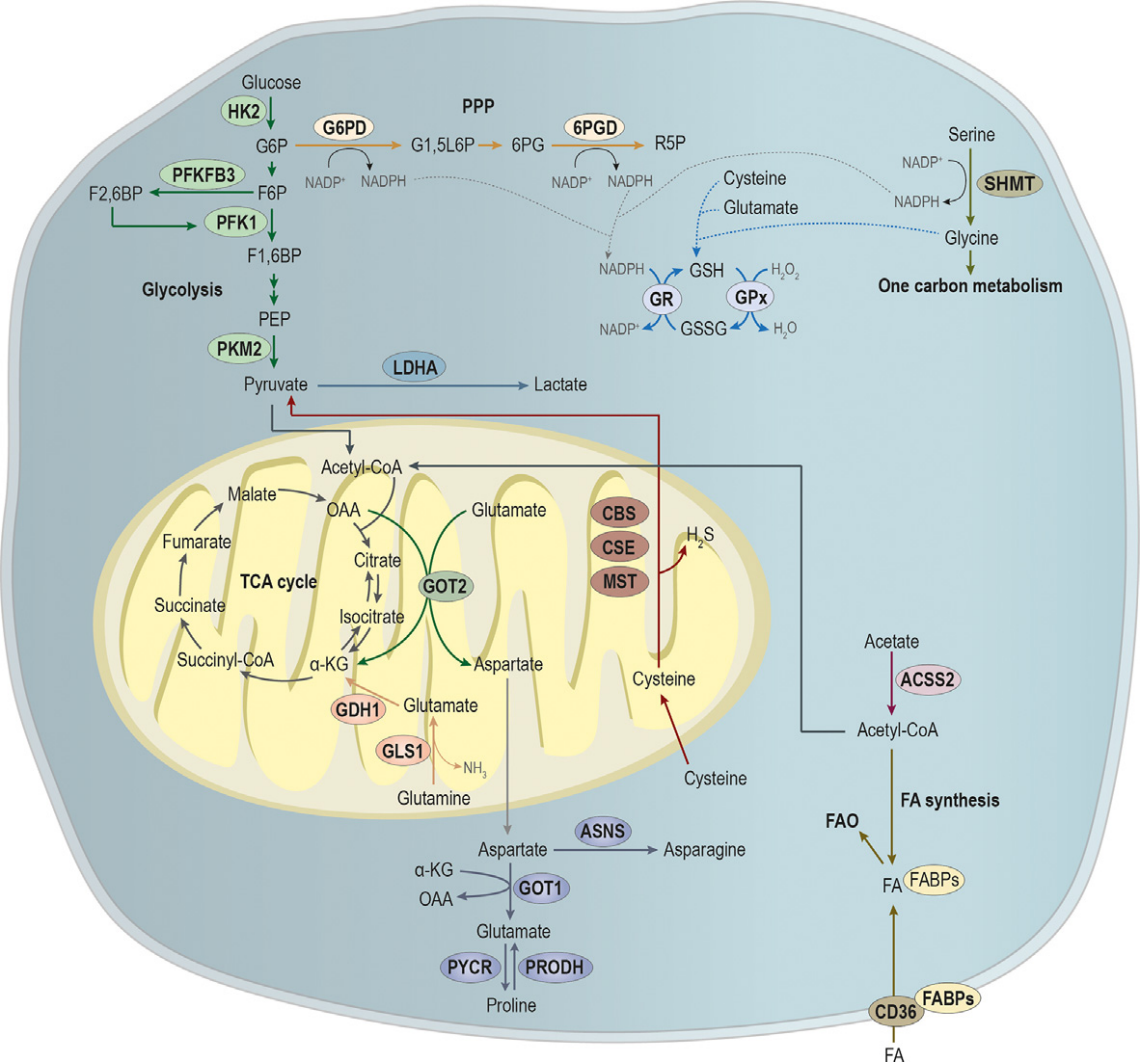
Main pathways involved in the metabolic remodeling of metastatic cancer cells, pivotal for energy and biomass production. Metastatic cancer cells are metabolically more plastic than non-metastatic cancer cells, because they need to survive in primary tumor microenvironment, and they must also prosper in the microenvironment of the metastasized organ. Glycolysis is a multi-step process, in which the expression of key glycolytic enzymes as hexokinase 2 (HK2) that converts glucose in glucose-6-phosphate (G6P), 6-phosphofructo-2-kinase/fructose-2,6-biphosphatase 3 (PFKFB3) that promotes the conversion of fructose-6-phosphate (F6P) in fructose-2,6-biphosphate (F2,6BP; an allosteric activator of the glycolytic enzyme phosphofructokinase 1 (PFK1)) and pyruvate by tumor M2-pyruvate kinase (PKM2) that synthesizes pyruvate from phosphoenolpyruvic acid (PEP) have been showed to be involved in metastases establishment. Pyruvate is an endpoint product of glycolysis that after its conversion into acetyl-CoA, which can alternatively be produced from acetate, under the action of acyl-coenzyme A synthase short-chain family member 2 (ACSS2), then it will supply the tricarboxylic acid (TCA) cycle or the synthesis of fatty acids (FA). Additionality, FA can be imported by CD36, being the FA pool deviated to FA b-oxidation (FAO). Glycolysis intermediate glucose-6-phosphate (G6P) can be diverged from glycolysis to supply phosphate pentose pathway (PPP) through the action of glucose-6-phosphate dehydrogenase (G6PD), catalyzing the conversion of G6P into glucono-1,5-lactone-6P (G1,5L6P) and generating nicotinamide adenine dinucleotide phosphate (NADPH). The 6-phosphogluconate dehydrogenase (6PGD) converts 6-phosphogluconate (6PG) into ribose-5-phosphate (R5P), concomitantly with NAPDH production. Amino acids metabolism is crucial in biosynthesis and bioenergetics. The serine and glycine syntheses and the one-carbon metabolism play a central role in cell metabolism. The action of serine hydroxymethyltransferase (SHMT) catalyzes the conversion of serine into glycine, generating NADPH. NAPDH will be essential for the reduction of glutathione disulfide (GSSG) into glutathione (GSH) by the action of glutathione reductase (GR). In turn, the oxidation of GSH in GSSG by glutathione peroxidase (GPx) is essential for the control of the redox state of metastasizing cancer cells. Moreover, cysteine, glutamate and glycine are used in GSH synthesis. Glutamine catabolism through the action of glutaminase 1 (GLS1) leads to the production of glutamate and ammonia (NH_3_). In turn, glutamate dehydrogenase 1 (GLDH1) converts glutamate into α-ketoglutarate (α-KG) that will fulfill TCA cycle. In mitochondria, glutamate is a target of glutamic-oxaloacetic transaminase 2 (GOT-2), producing α-ketoglutarate (α-KG) and aspartate, at the expense of oxaloacetate (OAA). In cytoplasm, the action of GOT-1, at the expense α-KG, leads to glutamate production that can be converted into proline by the action of proline dehydrogenase (PRODH). Moreover, pyrroline-5-carboxylate reductase (PYCR) catalyzes the inverse reaction, leading to the conversion of proline into glutamate. Aspartate catabolism by asparagine synthetase (ASNS) promotes asparagine synthesis. Cysteine in cytoplasm is essential for GSH synthesis while in mitochondria, its catabolism by the cystathionine-β-synthase (CBS) route; the cystathionine-γ-lyase (CSE) route and the CAT/GOT:3-mercaptopyruvate sulfurtransferase (MST) axis route leads to the production of pyruvate and hydrogen sulfide (H_2_S).

Over the last years, cancer metabolism gained an increasing research interest, since metabolic reprogramming was considered a hallmark of cancer (30, 31). For a tumor to be established, it requires a metabolic adaptation to the microenvironment, allowing the tumor to grow and thrive. Moreover, metastatic cells require further alterations to the metabolic profile, once they have to adapt and prosper in the microenvironment of the metastasized organ (32). In this section, we will explore metabolic alterations in the main pathways involved in energy and biomass production upon metastasis (**Figure 2**).

### Glycolysis

The enzymes hexokinase (HK) 2 and pyruvate kinase (PK) M2 are key enzymes in glycolysis as they define the beginning and ending of the pathway. HK2 catalyzes the first rate-limiting step of glycolysis, converting glucose to glucose-6-phosphate, which may be deviated into the phosphate pentose pathway (PPP); and PKM2 converts phosphoenolpyruvate to pyruvate, linking glycolysis to an enormous metabolic network, such as lactate, tricarboxylic acids, fatty acids and amino acids metabolism (33). Both enzymes have been associated with metastasis formation, since their expression is correlated with increased lactate production (34, 35), concomitant with enhanced motility and invasive capacity (20). Given the importance of these enzymes in glycolysis, their knockdown results in impaired glycolysis and decreased lactate levels, being this associated with decreased motility and invasion (34, 36). Zhang et al. described c-Src, a tyrosine kinase, as a regulator of both HK1 and HK2, increasing their catalytic activity by phosphorylation of their tyrosine kinase residues, and consequently enhancing glycolysis in HCT116 cell line (37). c-Src is constitutively activated or present in increased protein levels in several human cancers (38). Moreover, its phosphorylation and activation promotes *in vitro* cancer cells migration and invasion, and an increased formation of lung metastasis *in vivo*, in a process dependent on both HK1 and HK2 (37). In addition, silencing HK2 in neuroblastoma cells reduces the growth of xenograft tumors and the incidence of lung metastases in mice (39). This association seems to be related to the glycolysis-derived methylglyoxal, a metabolic by-product known to activate yes-associated protein (YAP) signaling in breast cancer cells, which is crucial in the upregulation of genes that promote cell proliferation (*e. g.* CTGF, CYR61, and ANKRD1, ERBB4, FOS, AP-1) (40, 41), inhibit apoptosis (*e. g.* Bcl-2 family members) (42, 43), and drive EMT (44). In fact, EMT requires YAP/TAZ (transcriptional co-activator with PDZ-binding motif) to trigger cancer stemness and metastasis (45–47).

In pancreatic cancer patients, the upregulation of PKM2 was related to advanced tumor stage and metastasis (48). The same was observed in colorectal and gastric cancer patients with advanced and metastatic disease, who were found to have augmented levels of PKM2 in their plasma (49). In early stage breast cancer patients, the levels of circulating miR-122, a negative regulator of PKM2, can be used to predict metastatic progression (50). The PKM2 regulatory dynamics by miR-122 seems to result from a crosstalk between cancer cells and non-malignant cells. Thereby expressing and secreting miR-122, breast cancer cells control the glucose consumption by non-malignant cells, which will uptake miR-122 and reduce PKM2 expression, and consequently glycolysis, in such way increasing glucose bioavailability to be used by cancer cells (51). Accordingly, the inhibition of miR-122 expression by cancer cells reduces the incidence of metastasis, whereas miR-122 overexpression increases the metastatic rate (51).

PFKFB3 (6-phosphofructo-2-kinase/fructose-2,6-biphosphatase 3) expression is increased in cancer and it acts as an allosteric activator of the glycolytic enzyme phosphofructokinase 1 (PFK1), stimulating glycolysis (52). Additionally, PFKFB3 activity is essential for cell proliferation, as it prompts the cell cycle progression from the G1 to S phase (52). PFKFB3 is also important in the metastatic cascade since its expression in endothelial cells, which are hyperglycolytic, promotes angiogenesis and the formation of cancer typical leaky vessels, favoring the intravasation and metastatic spread of cancer cells (53).

The increased rate of glycolysis observed in cancer results, in part, in the production of lactate. Although lactate does not stimulate primary tumor growth in an *in vivo* breast cancer model, it functions as a chemo-attractant *in vitro* and *in vivo* it increases lung metastasis formation by 10-fold (54). The lactate-induced migratory pattern was shown to depend on β1-integrin expression (55), correlating with the metastatic behavior in a variety of tumors (56). Moreover, β1-integrin interacts with monocarboxylate transporter 4 (MCT4), a transporter associated to lactate export, characteristic among metastatic tumors, whose expression is regulated by hypoxia-inducible factor 1-alpha (HIF-1α) (57). Both MCT4 and β1-integrin are present at the leading edge of epithelial migrating cells (58). Since the conformation of integrins is pH-sensitive (59, 60), the loss of MCT4 can modify integrin signaling, cell adhesion and migration (61). In the other hand, the changes in pH promoted by lactate stimulate the secretion and activation of hydrolases, including cathepsins and MMP-9 that are known to degrade ECM components and consequently decrease the physical barrier permissive for invasion (62). In particular, MMP-9 expression is correlated with survival and the development of metastases in breast, ovarian and prostate cancer (63–65). MMP-2 is also linked to metastasis, since its upregulation by TGF-β2 is stimulated by lactate and increases the migratory capacity of malignant glioma cells (66). The increased lactate production is deeply related to increased proliferation capacity of cancer cells and disease progression. Thus, high concentrations of lactate in biopsies from breast, colorectal, cervical, head and neck and lung cancers were proposed to be indicative of increased metastatic potential (67–70).

The increased expression of monocarboxylate transporter 1 (MCT1), another lactate transporter very well studied in cancer, is part of a pro-metastatic cell phenotype, being associated to the activation of NF-κβ pathway, which also regulates ECM degradation and EMT induction (71). Therefore, silencing or deleting MCT1 *in vivo* leads to the inhibition of cancer cell migration, invasion and metastases formation (71). Accordingly, in invasive bladder cell lines and in muscle-invasive bladder cancer specimens, it was found a correlation between EMT markers and high levels of lactate dehydrogenase A (LDHA) expression, the enzyme that mainly converts pyruvate into lactate in the end of glycolysis (72). Taken together, the expression of crucial glycolytic interplayers accompanies the phenotypical changes needed for EMT and metastasis.

### Pentose Phosphate Pathway

The pentose phosphate pathway (PPP) is an important part of glucose metabolism and, together with one-carbon metabolism (73), a major source of NADPH, which is critical for countering oxidative stress and maintain the metabolic flow (74). Thus, an upregulation of PPP could support the survival of detached metastasizing cells by neutralizing reactive oxygen species (ROS) through NADPH generation (75). The detachment of anchorage-dependent cells from the ECM contributes to a decreased uptake of glucose, which impairs PPP flux and induces high levels of ROS, leading to anoikis, a form of apoptotic cell death (76). However, the redox needs of metastatic cells depend on the microenvironment of the secondary organ targeted by metastasis. Breast cancer metastases are a good example, since brain metastases present high expression levels of phosphate dehydrogenase (G6PDH) and 6-phosphogluconolactonase, the enzymes from the oxidative branch of PPP, together with high levels of glutathione (GSH)-reductase and GSH S-transferase P (77), while bone metastases present the opposite pattern of these enzymes expression (78).

Mutated *K-Ras* has been described to drive the upregulation of GLUT1, cell survival in low glucose conditions, and direction of glycolytic metabolites down the non-oxidative branch of PPP (79). The NADPH generated by the PPP will provide the cell with ROS scavengers to control redox stress, particularly in detached cancer cells, which contributes to anoikis resistance. The oxidative and nonoxidative PPP were both found to be activated in metastatic renal cancer, as seen by an increase in the activity of G6PD and transketolase (TKT) (80), an enzyme that regulates ribose 5-phosphate levels and *de novo* nucleotide biosynthesis (81). However, in metastatic cancer cells, an increase in the nonoxidative PPP compared to the oxidative PPP was described due to an increase in TKT activity and TKT-like-1 (TKTL1) protein overexpression (80). This may fulfill the requirements for glycolytic intermediates and ribonucleotides in highly proliferative cancer cells. In agreement, TKT was also reported to be associated with metastasis of ovarian (82) and esophageal (83) cancer.

Different types of cancer present the upregulation of PPP in metastases in comparison to primary tumors, such as metastatic renal cell carcinoma (84), melanoma (73) and pancreatic cancer (85), in a way that involves not only metabolic adaptation but also epigenetic remodeling (73, 85). As mentioned before, the EMT controlling signaling pathways also regulate the glucose metabolism, therefore under oxidative and metabolic stress, Snail, a transcription factor that activates EMT (86), controls the flux of glucose *via* the PPP to allow cancer cell survival and enhanced metastatic potential (87).

Thus, genetic alterations in key metabolic genes accompanied by microenvironment-conditioned oxidative stress, inducing changes in the levels of ROS and NADPH, may contribute to cancer progression and metastasis.

### Mitochondrial Metabolism and Redox Balance

Several studies have shown that mitochondrial metabolism, in particular, oxidation of various nutrients (such as compounds derived from glutamine, glucose, and fatty acids) in the tricarboxylic acid (TCA) cycle, is linked to cancer invasion, being often upregulated in metastatic cancer cells (88, 89). Although the TCA cycle generates important biosynthetic intermediates that enable cancer cell proliferation, its principal role in metastasizing cells is likely to be bioenergetics (90). Metastasis requires that each disseminated cell has sufficient energy to migrate and invade, while avoiding induced cell death in circulation, attacks by the immune system, and even the hostile microenvironment of the secondary site in distant organs (91). Thus, the main obstacle to successful colonization of a distal site by circulating cancer cells is survival, rather than proliferation, since they might undergo anoikis, which is mainly induced by low ATP levels resulting from a decrease in glucose uptake in detached cells (92). Hence, metastatic cells can evade anoikis through the upregulation of the TCA cycle to cope with the decline in energy. Besides the one molecule of GTP produced in the TCA cycle, the precursor molecules NADH and FADH_2_ are also produced, which are used to generate ATP *via* the electron transport chain (ETC) and oxidative phosphorylation (OXPHOS) (93). Some studies showed that fatty acids β-oxidation feeds the TCA cycle with acetyl-CoA in order to generate ATP (94, 95). Moreover, it is important to note that biosynthesis is a process that consumes energy, requiring significant amounts of ATP to synthesize macromolecules.

Some intermediates of the TCA cycle may contribute to metastasis through epigenetic regulation. For instance, elevated levels of α-ketoglutarate (α-KG) were described to maintain the pluripotency of embryonic stem cells by global DNA hypomethylation (96). As EMT is regulated by epigenetic modulation and the EMT phenotype includes some stem-like features, α-KG may play a similar role in this context (97). Cancer cells usually present a deficient DNA demethylation ability, which is commonly associated with decreased α-KG levels and consequent alterations in α-KG-dependent epigenetic enzymes, as DNA demethylation ten-eleven translocation hydroxylases (TETs). In fact. Atlante *et al.*, found that targeting α-ketoglutarate dehydrogenase (KGDH), an important TCA enzyme responsible for the oxidative decarboxylation of α-KG to succinyl-CoA which promotes increased levels of α-KG, is able to recover TETs’ demethylation activity (98). TET expression stabilization by increased α-KG bioavailability is further associated with the induction of demethylation of miR-200 promoter and increased nitric oxide (NO) levels, the latter with known inhibitory effect in breast cancer progress (98, 99). KGDH inhibition impaired cell invasion ability *in vitro* and *in vivo* reduced migration and metastasis development in a metastatic breast cancer model (98).

TCA cycle intermediates succinate and fumarate tend to accumulate as a result of mutations in succinate dehydrogenase or fumarate hydratase, which lead to an upregulation of EMT-related genes (100, 101). Moreover, mutations in isocitrate dehydrogenases (IDHs) are also found to be related with EMT occurrence. IDHs are responsible for the decarboxylation of isocitrate to α-KG and are present in three isoforms: cytosolic IDH1 and mitochondrial IDH2 and IDH3. Mutations in IDH1 and IDH2 are described in several tumors, including gliomas, leukemia, breast and colorectal (102–106). These mutations in IDH hinder the enzyme to carry out its forward normal reaction, however, in a gain-of-function kind of way, it confers the enzyme the ability to subsequently convert αKG to 2-hydroxyglutarate (2-HG), an onco-metabolite that is found in high levels in patients presenting IDH mutations (107, 108). 2-HG was found to be an EMT inducer by affecting the ZEB1/miR-200 axis, through epigenetic modifications in histones, stimulating *ZEB1*, which is, by itself, a significant EMT regulator; and by promoting down-regulation in the miR200 family, also associated to the EMT phenotype (103, 104). Additionally, 2-HG was found to correlate with distant organ metastasis in colorectal cancer (104).

OXPHOS is frequently upregulated in highly metastatic cells in comparison to their less metastatic counterparts (109, 110), as well as in circulating tumor cells compared to primary tumor cells (111). For instance, through several cycles of adhesion impairment of a non-tumorigenic melanocyte cell line, Rodrigues *et al.* generated and selected stable clones displaying a metastatic phenotype. These metastatic cells released high amounts of glutamine catabolism-derived lactate, while displaying an upregulated OXPHOS dependent on glutaminase activity, with increased oxygen and succinate consumption and enhanced fatty acids oxidation, which contributed to an augment of ATP synthesis (109). Moreover, this was not accompanied by an increase in mitochondrial content or biogenesis, reinforcing the contribution of OXPHOS to the metastatic phenotype. Others also showed that a mammary epithelial cancer cell line exhibited enhanced OXPHOS, mitochondria biogenesis and respiration *via* the transcription coactivator peroxisome proliferator-activated receptor gamma, coactivator 1 alpha (PGC-1α) (111). Accordingly, the role of OXPHOS was also reinforced in another study, showing that melanoma cells presenting a more metastatic phenotype were characterized by an increased OXPHOS in comparison with melanocytes, and when OXPHOS was impaired with Elesclomol (a drug shown to alter the redox balance) no major effects were observed in glycolysis (110). It has been shown that total abolishment of OXPHOS inhibits metastasis but dysfunctional OXPHOS surprisingly augments the metastatic behavior of cancer cells, despite impaired respiration and energy production (112). This is in agreement with the beneficial effect of increased glycolysis rates in metastatic cells. Another plausible explanation may be the fact that OXPHOS produces ROS, which are essential to mitochondria-orchestrated cell signaling and adaptation to stress (113). Not only the activation and inhibition of mitochondrial biogenesis, but also the mitochondrial overload and dysfunction have been described to drive invasiveness of cancer cells (89, 111, 114), since it may activate the protein tyrosine kinases Src and Pyk2, known to drive cancer cells motility by remodeling cell-cell and cell-matrix interactions (89). Accordingly, the impairment of mitochondrial redox homeostasis and administration of a mitochondrial ROS scavenger has been shown to block cancer progression *in vivo*, including metastasis formation (89, 115). PGC1α was reported by LeBleu and colleagues as a metastatic sponsor by stimulating mitochondrial biogenesis and OXPHOS related genes, being expressed in circulating tumor cells, probably supporting dissemination. This suggests that PGC1α dysfunction may impact on the oxidative capacity of cancer cells probably by altering the ETC efficacy and ATP production capacity. Moreover, this study showed a significant correlation between PGC1α expression and the formation of distant metastases in human invasive breast cancer, associated with OXPHOS promotion and mitochondrial biogenesis (111). In prostate cancer, however, PGC1α was reported as a metastatic suppressor, preventing tumor growth and metastasis development through its oxidative metabolism-induced effects. Nevertheless, inhibiting PGC-1α may result in contradictory effects on cancer invasion, which could be related to the cancer cell of origin and genetic drivers (20, 111, 114, 116). Moreover, the expression of PGC-1α hinders prostate cancer progression by activating catabolism *via* estrogen-related receptor alpha (ERRα) (114). The same was observed in melanoma: PGC-1α blocks the invasive capacity of cancer cells, and by knocking down PGC-1α; invasiveness is rescued through the transcription factor 4 (TCF4)–focal adhesion kinase (FAK) signaling axis, that modulates cell–cell and cell–matrix interactions (116). These contradictory and cancer-specific observation suggest that metabolic rewiring may have different meanings in different cancer contexts, depending on cancer cell needs, genetic landscape and the overall tumor microenvironment (114).

Additionally, the oxidation of glutamine or fatty acids, important substitutes of glucose, has been associated with increased invasiveness and aggressiveness in breast, melanoma and ovarian cancer (95, 109, 117). These findings may be connected to cell motility programs as actin cytoskeleton remodeling and contraction processes. Accordingly, these processes require high energy demands and these can be fueled by mitochondrial-produced ATP (20).

Regarding mitochondrial metabolism of glutamine and glutamate, which will be depicted in the section *Amino Acid Metabolism*, a study showed that invasive breast cancer cells were able to secrete glutamate to induce the recycling of membrane type 1 MMP (MT1-MMP) through metabotropic glutamate receptor 3 (GRM3), thereby controlling the invasive capacity (118). Glutamine-derived glutamate is a component of GSH molecule to control the redox state, and an important source of α−KG to fuel the TCA cycle (32). The expression of glutamine synthase (GS), which synthesizes glutamine from glutamate and ammonia (119), is associated with the suppression of hepatocellular carcinoma metastasis (120), showing that the glutamine bioavailability may be part of the metabolic rewiring of metastatic cancer cells.

Cells that experience loss-of-attachment to the extracellular matrix show an inhibition of glucose uptake and catabolism, resulting in loss of cellular ATP. Successful intravasation and extravasation during metastasis entail that cancer cells produce or obtain more ATP through other ways (76, 121). Fatty acids β-oxidation is an energy-efficient way to generate ATP to fulfill the energy requirements of cancer cells (122). For instance, metastatic ovarian cancer cells catabolize lipids obtained from omental adipocytes to meet their high energy demands through β-oxidation, which allows them to survive the colonization process (123). Other examples of the involvement of β-oxidation in the metastatic process are further described in the section *Fatty Acid Metabolism*.

A well-balanced microenvironment is the key for metastasis and ROS maintenance and scavenging through antioxidants, which act as neutralizing-electron donors and are crucial for cancer cell survival. ROS levels increase occurs by several endogenous factors and mostly upon dysfunctional ETC resultant from problems in OXPHOS or excess TCA cycle activity, leading to increased mitochondrial byproducts of oxygen metabolism. However, cancer cells can use this event on their favor if these levels are not excessive and detoxifying options are available. Moderate ROS levels are associated with metastasis by inducing several mitochondrial signaling pathways (113, 124–126). Antioxidants agents can be categorized as enzymatic (as superoxide dismutase, catalase, thioredoxin peroxidase and GSH peroxidases) or non-enzymatic (as ascorbic acid and *N*-acetyl cysteine) (127). Superoxide dismutase, for example, has been reported as an invasion inducer, associating directly with increased metastasis, through VEGF signaling (128–131). Overall, this antioxidant agent partners with cancer cells, detoxifying an oxidative stress-rich microenvironment with the goal of supporting cancer cells proliferation and colonization capacity. ROS *per se*, when present in non-lethal concentrations, can regulate the activation of growth factors and integrins by different processes, for example, the regulation of dynamics in the cytoskeleton as a pro-metastatic agent (132). Moreover, it can also rewire metabolic pathways as ERK1/2 activation, which is upstream from STAT3 and HIF-1 (133).

Taken together, mitochondrial metabolism may act as a double-edged sword, since it may be anti- or pro-metastatic, depending on tumor context and on alterations in the activation of different signaling pathways and redox conditions.

### Amino Acid Metabolism

Amino acids are the building blocks of proteins, playing crucial roles on the dynamic and homeostasis maintenance in cell (patho)physiology. Much as the previous mentioned metabolic pathways, amino acid metabolism is responsible for the supply of intermediates, energy and other mediators, and share crossroads with several metabolic trails, thus interfering with downstream pathways, when altered.

Cancer cells exhibit unique demands, as for the specific amino acid dependency that correlate with their malignant phenotype. In fact, several amino acids have appeared to be in close relation to tumorigenesis and to the metastatic process (134). Both tyrosine and phenylalanine have long been studied within the metastatic context and its restriction has shown anti-tumor effects (135–137). Remarkably, Pelayo and colleagues reported that tyrosine and phenylalanine restriction affected the metastatic potential of cancer cells by impacting across the overall process of invasion *via* inhibition of the Ras/Rho pathway, the latest, *per se*, being an important signaling pathway for the binding of integrins to the ECM (138). Tyrosine was proposed as a possible therapy option in metastatic cancer treatment by Gueron *et al.* This metabolite showed anti-proliferative effects and impaired tumor growth in metastatic prostate cancer models, without known toxic effects. This work shows that tyrosine induced autophagy in PC3 cells, impairing STAT3/NFκB/Notch pathway. Moreover, tyrosine abolished the formation of metastasis in breast cancer models. However, this study also reveals phenylalanine as capable of reverting tyrosine effects (139).

Glutamine is an important non-essential amino acid, highly abundant in the bloodstream and of great preference of tumors, being, thus, present in low concentration in the latter (136, 137). Moreover, this amino acid is essential in tumor progression, given its role in interorgan ammonia transport maintenance among proliferating cells (140). Glutamine is the precursor of glutamate, an equally important amino acid across metabolism, displaying many functions. For this matter, we will focus on the glutamine-glutamate axis role as a supplier for TCA and for the production of GSH, a tripeptide composed by glutamate, cysteine and glycine, which is the main cellular ROS scavenger (75). Glutaminase 1 and 2 (GLS-1 and -2) are responsible for the conversion of glutamine into glutamate, which is subsequently converted by NADP(+)-dependent glutamate dehydrogenase 1 and 2 (GDH1 and -2) into α-KG. While GLS-1 expression is associated with ovarian cancer invasiveness, GDH1 expression is reportedly associated with colorectal cancer metastasis, both mainly by supplying the TCA and activating the signal transducer STAT3 (117, 141). STAT3 regulates EMT, cell migration and invasion, and is also indirectly related to hypoxia-related cancer metabolic remodeling, under the control of HIF-1α (142). Moreover, glutamate is also a substrate for glutamic-oxaloacetic transaminase 1 and 2 (GOT-1 and -2), resulting α-KG and aspartate, at the expense of oxaloacetate (143). GOT-2 is directly regulated by STAT3 in lymphoma (144), but few studies have focused on the role of any form of GOT in metastasis. However, given the pro-metastatic roles of STAT3 (145–149), GOT-2 may be related to the pro-metastatic effects of STAT3. Moreover, GOT-1 and -2 also display a cysteine aminotransferase (CAT) activity, catalyzing the transamination of cysteine to 3-mercaptopyruvate and glutamate (143). Cysteine is an amino acid, which has three main associated catabolic routes: the cystathionine-β-synthase (CBS); the cystathionine-γ-lyase (CSE) and the CAT/GOT:3-mercaptopyruvate sulfurtransferase (MST) axis; all of them producing H_2_S concomitantly with cysteine degradation. H_2_S is a gasotransmitter with both physiologic and pathophysiologic effects, depending on its availability in the microenvironment. Increased H_2_S generation is reported as a tumor bioenergetics stimulator, leading to increased ATP production by OXPHOS and glycolysis. Moreover, it is also reported as a neoangiogenesis promoter *via* PI3K/Akt and MAPK pathways, a chemoresistance inducer and it is, by itself a powerful antioxidant (150, 151). Wang et al. reported that induced H_2_S secretion by increased expression of *CTH* (gene encoding CSE) led to activation of IL-1β/NF-kβ pathway, which directly correlated with increased prostate cancer cell invasion. This study further indicates that knocking down *CTH* decreased metastasis, an effect that, as expected, was reverted by overexpressing *CTH*, indicating a pro-tumoral and pro-metastatic role of cysteine catabolism and H_2_S production in prostate cancer (152). Likewise, CBS has been reported to promote similar effects. Bhattacharyya and colleagues showed that CBS silencing had inhibitory effects on ovarian cancer cells proliferation, in peritoneal metastatic development and in resistance to cisplatin (153). Alongside, Wang et al. reported that CBS overexpression in hepatocellular carcinoma was associated with lack of sensitivity to doxorubicin (154). The downregulation of these enzymes consequently promotes a decrease in GSH and H_2_S production, important regulators of oxidative stress, and inhibits pathways such as NF-κB, relevant in cancer metastasis (153, 154).

The expression of asparagine synthetase (ASNS), the enzyme that generates asparagine from aspartate, is further reported as being enhanced in primary tumors and correlated with metastatic relapse (155). ASNS knock down or dietary asparagine restriction reduced metastasis and cancer progression while increased dietary asparagine or overexpression of asparagine synthetase drove metastatic progression (155). Asparagine stimulates the expression of GS, which is an important controller of glutamine requirements (119). Changing the availability of asparagine influenced the invasive potential of cancer cells through the increased expression of EMT genes (155). An interconnective regulation of asparagine and glutamine metabolism dependent on these amino acids’ availability is stated to be responsible for the control of cancer cells proliferation, evasion to apoptosis, migration/invasion and metastasis (156).

Serine and glycine have a tight link: serine hydroxymethyltransferases can reversibly convert serine into glycine in the context of the one-carbon metabolism. This conversion releases NADPH, and thus, can be beneficial in a metastatic context by balancing microenvironment redox and protecting cancer cells from oxidative stress, which is reinforced by the role of glycine as a component of GSH. These amino acids are additionally reported as epigenetic modulators in cancer cells through the contribution for the one-carbon metabolism and the synthesis of intermediates to supply DNA and RNA methylation (75, 157).

Proline has been described as a relevant amino acid, playing a role in the different steps of cancer progression and metastasis, mainly through the proline metabolic cycle dependent on NADPH (158). In breast cancer, increased levels of proline *de novo* synthesis coincide with metastatic cellular phenotype (159). Accordingly, studies suggest that proline metabolism, through proline dehydrogenase (PRODH) and proline dehydrogenase reductase (PYCR), is associated with breast cancer metastasis, being upregulated in metastases compared to primary breast cancer tumors (160, 161). Actually, in proline catabolism, PRODH activity generates ROS, which are able to promote metastasis through several mechanisms, such as upregulation of glycolysis (through HIF1-α), activation of several signaling cascades involved in survival (such as the MAPK, NF-kB, PI3K), induction of EMT (*e.g.* increase vimentin expression) and induction of angiogenesis (162). Moreover, the resultant product of proline oxidation pyrroline-5-carboxylate (P5C) can be converted into glutamate by pyrroline-5-carboxylate dehydrogenase (P5CDH), which can then be converted into α-KG, fueling the TCA cycle (163, 164). Therefore, proline can contribute for metastasis with ATP, ROS and the synthesis of other macromolecules.

Furthermore, proline was described as an epigenetic regulator in embryonic stem cells, leading to a migratory and invasive mesenchymal phenotype, possibly by interfering with methylation status, thus modulating gene expression (165). Comes and colleagues showed that L-proline is able to induce an embryonic-stem-cell-to-mesenchymal-like transition by embryonic stem cell transcriptome rewiring towards a motile, invasive and highly migratory pluripotent phenotype. This work shows that increased methylation levels in H3K9 and H3K36 seem to underly this remodeling, being the methylation status a key in the balance of this switch. In line with this, L-proline withdrawal or ascorbic acid (vitamin C) addition, a known cofactor for H3K9 jumonji demethylases (166) with regulatory roles in stem cell differentiation and somatic cell reprogramming (167, 168), reverts this process escorted by H3K9 and H3K36 demethylation (165). Moreover, vitamin C was also associated with pro-oxidant effects, *via* high ROS production (169), and while specific doses are able to stop cancer cell glycolysis and affect metastasis (170), recent studies have shown a role in metastasis promotion, being able to regulate cancer stem cells and affect extracellular matrix (171).

### Fatty Acid Metabolism

Nowadays, it is well known that fatty acid synthesis and uptake plus β-oxidation are associated with cancer progression. Both fatty acid uptake and accumulation have been shown to augment the invasiveness, migration, and progression of different types of cancer, such as liver and breast carcinomas (172, 173). Particularly, hepatocellular carcinoma progression was correlated with CD36/fatty acid translocase and high free fatty acid levels *via* induction of EMT, which was promoted by TGF-β and Wnt signaling (172). Fatty acid transport protein 1 (FATP1), a member of the FATP/SLC27 protein family, which increases the cellular uptake of long-chain fatty acids (174), has been linked to cancer progression (175) and is also likely to be involved in metastasis. Considering breast cancer patients and using data from the TCGA database, it was observed that metastases expressed higher levels of *FATP1/SLC16A1* when comparing to normal breast tissue (175). In addition, in melanoma patients, the expression of FATP1 was found to be higher in subcutaneous metastases when compared to primary tumors (176).

Fatty acids mobilized from lipid stores may be degraded in the mitochondria through β-oxidation to provide energy when necessary (177). In the β-oxidation pathway, acyl-CoAs are cyclically dehydrogenated, hydrated, and decarboxylated, leading to the progressive shortening of the fatty acid, together with the generation of NADH and FADH_2_ and acetyl-CoA (178). NADH and FADH_2_ will be used for ATP production in the ETC, and acetyl-CoA can enter the TCA cycle (178). Besides providing energy when glucose becomes limiting, β-oxidation also controls the oxidative stress by increasing the intracellular levels of NADPH (179). β-oxidation was reported as crucial for the survival of cells from solid tumors when undergoing loss-of-attachment, which leads to anoikis or cell death due to oxidative stress (121, 180). Enhanced β-oxidation has been described in metastatic breast cancer (75, 181), and in metastatic triple negative breast cancer (TNBC) the knock down of carnitine palmitoyltransferases, important elements in the carnitine shuttle of fatty acids into the mitochondria, led to the inhibition of the c-Src proto-oncogene and Src-mediated metastasis (75, 94). Halldorsson et al., performed metabolomics profiling of a breast epithelial cell line and its EMT derived mesenchymal variant and showed that β-oxidation, driven by peroxisome proliferator-activated receptor (PPAR), fueled the mesenchymal phenotype while glycolysis and OXPHOS were more active in the epithelial phenotype (182). Accordingly, an upregulation of β-oxidation was observed in colon cancer cell co-cultured with adipocytes and it was linked with EMT induction as indicated by reduced E-cadherin but increased vimentin expression (183, 184).

Fatty acid synthase (FASN) is the enzyme involved in endogenous fatty acid synthesis (185), which is crucial for the formation of membrane lipids and sustains redox equilibrium and the relative levels of saturated and unsaturated fatty acids in cancer cells (185). FASN has been shown to play a role in tumor invasion and metastasis, and FASN inhibition or knockdown reduced liver metastasis of colorectal cancer (186). The inhibition of FASN expression abolished the invasion and migration of HCC cells, demonstrating the contribution of FASN to malignant hepatocellular carcinoma metastasis (187). Additionally, FASN expression induced peritoneal metastasis of ovarian cancer through the induction of EMT (188).

Acetyl-CoA can be synthesized in different metabolic pathways (32, 189) and it is an important donor for protein and histone acetylation (190), as well as the main compound initiating the TCA cycle, the building block in fatty acid synthesis and the final product of fatty acids β-oxidation. Inhibiting the lipogenic enzyme acetyl-CoA carboxylase 1 (ACC1) results in the accumulation of acetyl-CoA, which activates an EMT program through Smad2 transcription factor acetylation and subsequently induction of breast cancer cell invasion and metastasis (191).

Acetyl-CoA can also be synthesized from acetate under the action of acetyl-CoA synthetase 2 (ACSS2), being further used in fatty acids synthesis and TCA cycle. ACSS2 silencing leads to deacetylation of HIF-2α during hypoxia, which in turn results in HIF-2α-dependent EMT activation (192). These findings indicating that acetyl-CoA favors metastasis are in agreement with the results obtained in patient-derived xenograft melanoma models, showing that metastatic tumors presented high levels of metabolites related to histone methylation, including acetyl-CoA (193). Moreover, in these models, the inhibition of histone methylation blunted invasiveness and metastatic spread (193), resembling the inhibition of acetyl-CoA synthesis and showing again that the epigenetic remodeling needed for the display of the metastatic phenotype is tightly related to metabolic rewiring.

## From Primary Tumor to Metastasis: Metabolic Comparison

Over the last years, cancer metabolism gained an increasing research interest, though this was mainly focused on the primary tumor, while little is known about the metabolic remodeling that occurs during metastasis. Different organs in the human body present characteristic metabolic features (194), thus it is evident that metastatic subclones exhibit different phenotypes when compared to the primary tumor, according to the particular microenvironment of the organ. The deepening of this knowledge and the extensive characterization of the distinction between primary tumor and metastases metabolism is crucial for the development of new therapeutic strategies.

Dupuy et al. used a murine nonmetastatic breast cancer cell line and compared its metabolic profile with metastatic cell populations from the same parental tumor (195). The metastatic subclones were characterized by an increased utilization of pyruvate and glucose, as well as a greater oxidative capacity, resulting in increased glycolysis and OXPHOS. In this study, distinct cell populations that preferentially colonize the bone, lung, or liver presented characteristic metabolic changes, mainly regarding glucose utilization. While bone and lung metastasis engaged OXPHOS, glycolysis-dependent metabolic strategies were favored by liver-metastatic cells, *via* HIF-1α and PDK1. Moreover, bone and lung metastatic cells displayed increased glutamine uptake, which reflected in increased levels of glutamine-derived citrate and succinate, TCA cycle intermediates. These findings are of extreme importance since the understanding of these divergent metabolic profiles may contribute to the development of targeted therapies. Since then, several articles reinforced the importance of this comparison. Li and colleagues showed an upregulation of mitochondrial serine and one-carbon metabolism in metastatic subclones of TNBC cell line (MDA-MB-231), comparing to the nonmetastatic cells (196). This pathway is involved in proliferation, through *de novo* purine synthesis (197), and its impairment suppressed proliferation *in vitro* and impaired the growth of lung metastases in mice models, being correlated with poor survival of human breast cancer patients (196).

Consistent with these results, distinct metabolic profiles were revealed between non-metastatic and metastatic cells of human R-18 melanoma cell line (established from a xenograft model) by proton high resolution magic angle spinning magnetic resonance spectroscopy (^1^H-HR-MAS-MRS) (198). The metastatic tumors expressed higher levels of phosphocholine, creatine, and glycine, while lower levels of lactate were observed. These alterations in creatine and choline metabolism were consistent with a similar study using a B16F10 murine melanoma cell line, being these metabolites important for tumor progression and the development of liver and lung metastases (199). Moreover, while there is substantial evidence that the primary tumor is characterized by high lactate levels, favoring the release of metastatic cells, lactate production by metastases is not well stablished. For instance, Xu and colleagues used a breast carcinoma (MDA-MB-231) xenograft model and by using hyperpolarized ^13^C-pyruvate NMR spectroscopy found out that less metastatic breast tumors produced more lactate than the highly metastatic tumors (200). On the other side, Lemma et al. observed the release of large amounts of lactate from bone metastatic breast cancer cells when compared with non-osteotropic ones, promoting the formation of osteolytic lesions (201).

The wide variety of metabolic pathways supporting cancer invasion makes it challenging to design new effective therapeutic approaches. Besides, cancer cells dissemination to distant organs may be an early event that may occur before the initial diagnosis, thus the time frame for treatment targeting cancer invasiveness will be limited (202). Focusing on the metabolic changes that take place in primary tumors will likely be more useful to predict metastatic risk.

### Metabolic Targeting Encloses the Success of the Treatment of Metastatic Disease

**Figure 3:**
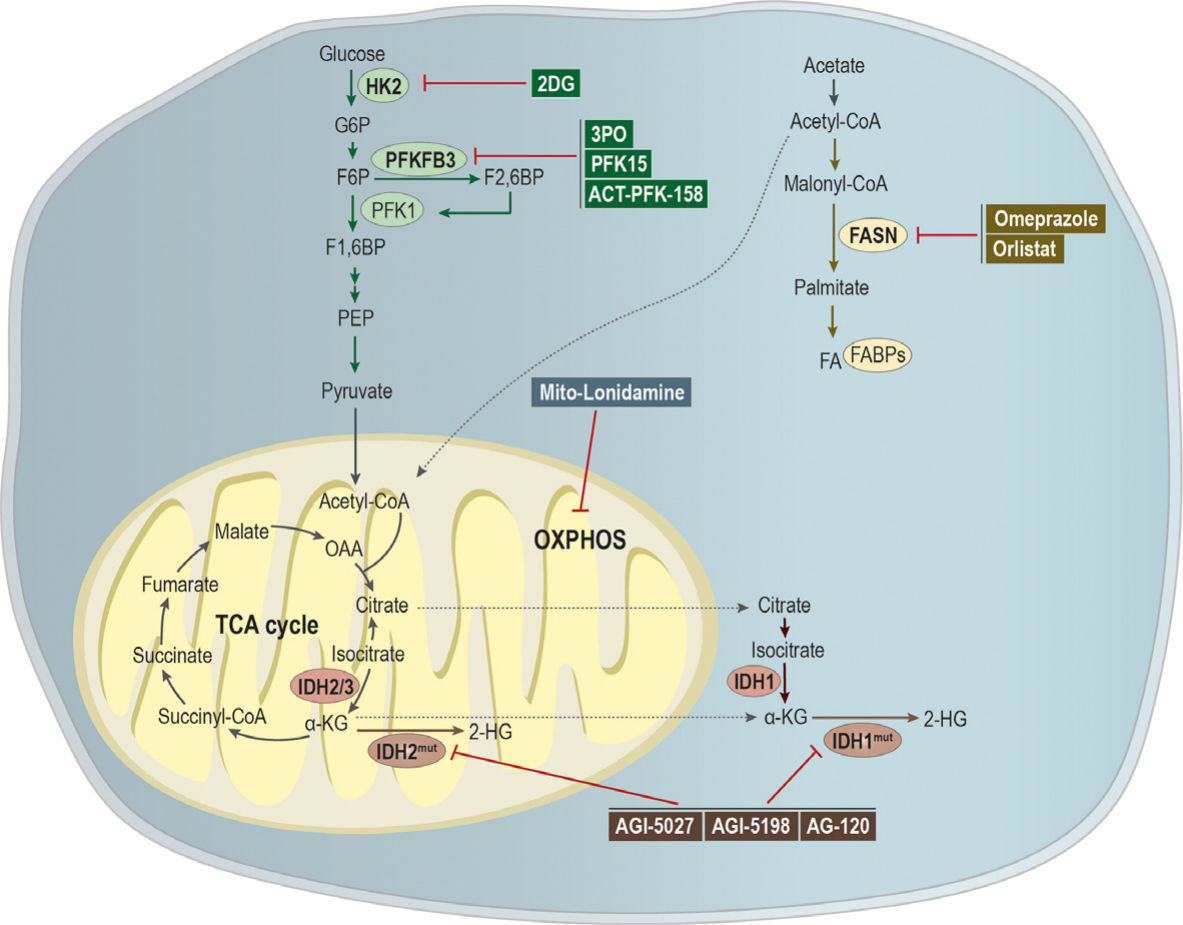
Targeting metabolic pathways as a putative strategy to tackle metastatic cancer. Glycolytic inhibitors have been shown as being a promising application for the treatment of metastatic cancer. The compound 2-deoxy-d-glucose (2-DG) acts as an inhibitor of hexokinase 2 (HK-2), while 3PO, 1-(4-pyridinyl)-3-(2-quinolinyl)-2-propen-1-one (PFK15) and ACT-PFK-158 were designed to inhibit the activity of PFKFB3. A modified version of lonidamine was developed to inhibit OXPHOS and mitochondrial bioenergetics. Mutations in isocitrate dehydrogenases 1/2 (IDH 1/2) leads to the generation of the oncometabolite 2-hydroxyglutarate (2-HG), being the development of IDH^mut^ inhibitors, as AGI-5027, AGI-5198, and AG-120, an attempt to abrogate 2-HG production. Moreover, omeprazole and orlistat, inhibitors of fatty acid synthase (FASN), showed a promising clinical applicability in the treatment of metastatic cancer.

Here we reviewed the impact of metabolism in metastatic cell characteristics, being this metabolic plasticity essential during cancer progression. Tumor cells have the capacity to use metabolites from the microenvironment for its own benefit, thus coping with the challenging conditions faced during the metastatic cascade (**Figure 3**). Therefore, targeting the metabolism of metastases could be a good strategy to tackle metastatic cancer.

Since glycolysis is upregulated in metastases, the use of inhibitors for key enzymes could be effective. Cheng and colleagues modified lonidamine, a HK2 inhibitor with limited efficacy, to mitochondria-targeted mito-lonidamine. This new inhibitor was more potent, able to inhibit OXPHOS and mitochondrial energetics in lung cancer cells, inducing autophagy-related cell death, decreasing the viability, growth, and brain metastasis of lung cancer xenograft mice models (203), without causing toxicity. Another HK2 inhibitor is 2-deoxy-d-glucose (2-DG) (204), a non-metabolizing synthetic glucose analog able to abolish ATP generation (205, 206). This analog is able to cross the brain-blood barrier, being a good candidate to target brain metastases (207). Therefore, a phase I dose-escalation trial (NCT00096707) evaluated the effect of 2-DG alone or combined with docetaxel in patients with histologically or cytologically confirmed locally advanced or metastatic solid tumors. A potential application as an adjuvant therapy was found, although its clinical use implies several side effects (208). Nevertheless, a study showed that 2-DG treatment resulted in effective migration and invasion inhibition of an invasive subclone of a TNBC cell line, through targeting glycolysis (209). Therefore, blocking invasiveness can be a new strategy to target tumor metastasis. Moreover, 2-DG treatment also resulted in decreased proliferation, colony formation, migration and invasion, and decreased expression of EMT-related genes, including quinone oxidoreductase-1-induced vimentin, Snail, Slug and Twist, and upregulation of E-cadherin (210).

Another glycolysis key enzyme is PFKFB3 and its blockage with the phosphatase inhibitor 3PO in endothelial cells resulted in tumor vessel normalization and impaired metastasis (53, 211). This enzyme is usually overexpressed in cancer, and its inhibition with 1-(4-pyridinyl)-3-(2-quinolinyl)-2-propen-1-one (PFK15) blocked glycolysis and suppressed cell proliferation, motility and induced apoptosis of head and neck squamous cell carcinoma cells (212). Also, *in vivo* data from metastatic niche models showed that PFK15 decreased lung metastases and extended life expectancy in mice (212). Currently, a derivative of 3PO, ACT-PFK-158, is under phase I clinical trial (NCT02044861) for advanced solid malignancies, as a monotherapy (213).

As mentioned above, IDH 1/2 are enzymes involved in the TCA cycle. When these enzymes are mutated, they use α-KG as a substrate to produce the oncometabolite 2-HG (214). Several clinical trials have been pursuing a strategy to inhibit 2-HG production. Several studies using IDH1^R132C^ mutant inhibitors, such as AGI-5027 (215) and AGI-5198 (216), sustain a potential use to target metastasis, once they affect pathways involved in the metastatic cascade, such as mTOR pathway (217). Several clinical trials are currently active (NCT02073994, NCT02989857, NCT02074839, NCT04195555) to evaluate AG-120 (IDH-mutant inhibitor) efficacy and safety in several cancer types, being this already FDA-approved for IDH-mutant relapsed or refractory acute myeloid leukemia (218, 219). However, this therapy can be a double-edge sword, since IDH mutant tumors are less proliferative and invasive than IDH wild-type tumors (140).

Jin and colleagues showed that omeprazole inhibited MDA-MB-231 breast cancer cell invasion *in vitro* and lung metastasis in a mouse model, alongside with decreased expression of two prometastatic genes, *MMP-9* and C-X-C chemokine receptor 4 (*CXCR4*) (220). It is known that FASN is inhibited by omeprazole (221, 222) and, currently, a phase 2 clinical trial is evaluating the use of omeprazole to improve the efficacy of neoadjuvant chemotherapy in breast cancer (NCT02595372). Moreover, orlistat, an irreversible inhibitor of FASN, was shown to reduce tumor lung metastases (53.4%) in C57BL/6 mice subcutaneously injected with melanoma B16-F10 cells (223). Interrupting the mechanisms of lipid uptake through the pharmacological inhibition of FATP1 with arylpiperazines (224) represents a promising therapeutic strategy for breast cancer and melanoma, as this protein is overexpressed in these tumors (175, 176). Although the effect of arylpiperazines on invasion and metastasis has not been studied yet, the inhibition of FATP1 with arylpiperazines interfered with the uptake of fatty acids and cell viability of breast cancer cells (175).

More studies are needed to expand the knowledge but mapping the alterations upon the metabolic drift cancer cells undergo, enabling metastasis, is a profitable field to identify new targets towards personalized medicine in cancer treatment.

## Highlights

Even though metastasis is a widely researched field, there is still a lot to understand about the metastatic processes, the genetic and metabolic profile, and essentially what incites cancer cells to metastasize. There is a wide variety of metabolic alterations related to cancer metastasis, either in the primary tumor or in the metastatic tumor. Defining the metabolic profile in each step of the metastatic cascade would provide clues to the requirements needed for a metabolism-based therapy. Although several preclinical studies have been made, there is a lack of clinical trials evaluating the effect of several therapies in metastatic cancer, aiming to incorporate them into the standard of care. In the future, taking the tumor microenvironment into consideration will also contribute to the identification of new molecular targets to suppress metabolic crosstalk pivotal to facilitate the adaptive process of cancer cells to different microenvironments and consequently to sustain their survival.

## Author Contributions

AH, FM, and CM wrote the first draft of the paper. FL-C discussed and revised the paper. JS supervised, discussed, and revised the paper. All authors contributed to the article and approved the submitted version.

## Funding

The research group was funded by IPOLFG EPE and by iNOVA4Health (UID/Multi/04462/2019) a program financially supported by *Fundação para a Ciência e Tecnologia (FCT)/Ministério da Educação e Ciência*, through national funds. All the fellowships were funded by FCT: AH (SFRH/BD/148441/2019), FM (2020.04780.BD), CM (2020.06956.BD), FL-C (PD/BD/128337/2017). 

## Conflict of Interest

The authors declare that the research was conducted in the absence of any commercial or financial relationships that could be construed as a potential conflict of interest.
